# Associations Between Multimorbidity, Polypharmacy and Oral Conditions in Older Adults: A Systematic Review and Meta‐Analysis

**DOI:** 10.1111/ger.70065

**Published:** 2026-05-17

**Authors:** Kittipit Srisanoi, Sabrina Maniewicz, Claudio Rodrigues Leles, Gerry McKenna, Frauke Müller, Murali Srinivasan

**Affiliations:** ^1^ Clinic of General, Special Care, and Geriatric Dentistry Center for Dental Medicine, University of Zurich Zurich Switzerland; ^2^ Faculty of Dentistry Khon Kaen University Khon Kaen Thailand; ^3^ Division of Gerodontology and Removable Prosthodontics University Clinics of Dental Medicine, University of Geneva Geneva Switzerland; ^4^ Department of Prevention and Oral Rehabilitation, School of Dentistry Federal University of Goias Goainia Brazil; ^5^ Center of Public Health Queen's University of Belfast Institute of Clinical Sciences Belfast UK; ^6^ Center of Excellence in Precision Medicine and Digital Health, International Program in Geriatric Dentistry and Special Patients Care, Faculty of Dentistry Chulalongkorn University Bangkok Thailand

**Keywords:** geriatric dentistry, meta‐analysis, multimorbidity, older adults, polypharmacy, systematic review

## Abstract

**Objective:**

This systematic review and meta‐analysis evaluated the relationship between multimorbidity and polypharmacy on the oral health in older adults (aged 75 years or older). The PECO focus question set for this study was, ‘What is the occurrence, association and impact of multimorbidity and polypharmacy on the oral conditions and its management in older adults?’.

**Methods:**

Electronic searches of MEDLINE (PubMed), Embase, and the Cochrane Library were conducted using the PECO (Population Exposure Comparison Outcome) framework. About 3433 studies published until April of 2025 were screened. Risk of bias was assessed using the Newcastle–Ottawa Scale. All eligible studies were descriptively analysed; studies that provided sufficient information were meta‐analysed.

**Results:**

Twenty‐three studies [21 cross‐sectional and 2 cohort studies] were included. Polypharmacy showed a significant association with dry mouth, as demonstrated by the pooled analysis (OR = 2.05; 95% CI: 1.31–3.22), along with consistent findings linking a higher number of medications to dry mouth and hyposalivation. Poor oral health assessment tool (OHAT) scores were also consistently associated with polypharmacy. In contrast, findings regarding multimorbidity/comorbidity were not consistently associated with dry mouth or with poor OHAT scores. Both exposures showed inconsistent associations with number of teeth, edentulism, and oral function. Quality assessment indicated that the included studies were predominantly at low risk of bias.

**Conclusions:**

Polypharmacy may impact on dry mouth and hyposalivation in older adults, potentially independent of multimorbidity. However, the impact of multimorbidity and polypharmacy on other oral conditions remains unclear. Further high‐quality evidence, particularly from longitudinal studies, is needed to clarify these relationships and establish causal associations.

**Trial Registration:**

PROSPERO Registration: CRD420251003698

## 1 Introduction

The global population is aging rapidly, and advances in medical care and facilities have enabled people to live longer lives. However, increased longevity is often accompanied by the accumulation of multiple chronic diseases and the need for numerous medications, presenting significant health challenges among older adults [1]. Two of the most prevalent and impactful issues in this demographic are multimorbidity and polypharmacy, both of which are associated with adverse health outcomes, such as reduced quality of life and frailty [2, 3].

The World Health Organization (WHO) defines multimorbidity as the presence of two or more long‐term chronic health conditions, encompassing physical and mental disorders, ongoing conditions, symptom complexes, sensory impairments, and substance misuse [4]. Recent estimates indicate a pooled prevalence of 42.4%, with substantially higher rates among older adults [5]. While comorbidity refers to the presence of additional conditions in relation to a specific index disease [6]. The Charlson Comorbidity Index (CCI) includes 17 conditions weighted according to one‐year mortality risk [7], whereas the Functional Comorbidity Index (FCI) includes 18 conditions, assigns equal weight to each, and emphasises conditions affecting physical function, such as visual impairment, osteoporosis, and arthritis [8].

Polypharmacy, commonly defined as the concurrent use of five or more medications, including prescription medications, over‐the‐counter drugs, and traditional or complementary medicines used by a patient [4]. Although polypharmacy is often associated with multimorbidity, not all older adults with multiple conditions are prescribed multiple medications [9]. The prevalence of polypharmacy is estimated to be approximately 40% among older adults worldwide, with higher rates observed in those aged 70 years and older, particularly among individuals with frailty, cognitive impairment, and frequent healthcare utilisation [10]. Moreover, 32.3% of care‐home residents experience excessive polypharmacy, defined as the use of 10 or more medications [11].

Research has extensively examined individual diseases or medications associated with oral conditions, whereas the effects of multimorbidity and polypharmacy remain an emerging area of investigation. Associations with common oral health problems in older adults, such as periodontal disease, dental caries, tooth loss and dry mouth, have been reported. However, the variability and cumulative effects have not been systematically explored. Therefore, understanding how multimorbidity and polypharmacy influence oral conditions is critical for developing preventive and therapeutic strategies that support this vulnerable population. This systematic review and meta‐analysis aimed to synthesise current evidence on the occurrence, associations, and impact of multimorbidity and polypharmacy on oral conditions and their management in older adults. Based on the aim of this syetematic review, the Population Exposure Comparison Outcome (PECO) focus question set was, “What is the occurrence, association and impact of multimorbidity and polypharmacy on the oral conditions and its management in older adults?”

## 2 Materials and Methods

This systematic review and meta‐analysis were carried out and reported following the PRISMA (Preferred Reporting Items for Systematic Reviews and Meta‐Analyses) guidelines [12, 13]. The completed PRISMA checklist is provided as supplementary data (Data S1). The systematic review protocol was registered with PROSPERO (the International Prospective Register of Systematic Reviews) under the registration number CRD420251003698.

### 2.1 Eligibility Criteria and Information Sources

The complete list of inclusion and exclusion criteria for this systematic review, along with the sources of information used to identify relevant records, are detailed in Table 1. The final search was performed on April 17, 2025 with an updated search conducted on December 22, 2025.

**TABLE 1:** Population Exposure Comparison Outcome (PECO) table showing the focus question, inclusion criteria, information sources, search terms and the search strategy applied for this systematic review.

Focus question	What is the occurrence, association and impact of multimorbidity and polypharmacy on the oral conditions and its management in older adults?
PROSPERO	CRD420251003698
Database search	
Database search	MEDLINE (PubMed), Embase, the Cochrane Library
Supplementary hand search	
References	Included articles and identified reviews
Others	Online search engines (Google)
Grey literature search	
Contact with experts	Authors of included articles. Researchers with a known interest in gerodontology research
Selection process	
Inclusion criteria	Studies reporting on the association between multimorbidity and/or polypharmacy and oral conditions, as well as their management in older adults aged 75 or older (or with mean age of participants ≥ 75 years). Sample size ≥ 10 participants per study group
Exclusion criteria	Review articles, study protocols, abstracts only. Insufficient participant information, and no response from investigators when clarification was sought. Previous investigations (double publications) reporting on the same patient population (excluded, but retained for reference). In such cases only the most recent report was included.
Contact with authors	Research that potentially met the inclusion criteria, but full text article was not available. Research that potentially met the inclusion criteria, but data was incomplete, insufficient or unclear. Research identified through grey literature search
Search terms	
Population	#1 [MeSH]: ‘Aged’ OR ‘Aged, 80 and over’. #2 [All fields]: ‘Elderly’ OR ‘Older Adult*’ OR ‘Elderly Adult*’
Exposure	#3 [MeSH]: ‘Comorbidity’ OR ‘Cardiovascular Diseases’ OR ‘Hematologic Diseases’ OR ‘Metabolic Diseases’ OR ‘Neurocognitive Disorders’ OR ‘Cerebrovascular Disorders’ OR ‘Rheumatic Diseases’ OR ‘Depressive Disorder’ OR ‘Kidney Diseases’ OR ‘Liver Diseases’ OR ‘Respiratory Tract Diseases’ OR ‘Immune System Diseases’ OR ‘Gastrointestinal Diseases’ OR ‘Polypharmacy’ OR ‘Hypoglycemic Agents’ OR ‘Cardiovascular Agents’ OR ‘Hematologic Agents’ OR ‘Bone Density Conservation Agents’ OR ‘Central Nervous System Agents’ OR ‘Immunosuppressive Agents’ OR ‘Adrenal Cortex Hormone’. #4 [Title/Abstract]: ‘Systemic Disease*’ OR ‘Multiple Medication*’ OR ‘Diabetes’ OR ‘Hypertension’ OR ‘Heart Disease*’
Comparison	No search term
Outcome	#5 [MeSH]: ‘Oral Health’ OR ‘Dental Health Surveys’ OR ‘Mouth Diseases’ OR ‘Jaw, Edentulous’. #6 [All fields]: ‘Oral health Status’ OR ‘DMF‐T’ OR ‘Gingival Index’ OR ‘Plaque Index’ OR ‘Periodontal Status’ OR ‘Number of Teeth’
Filters	No filters applied
Search Builder	(#1 OR #2) AND (#3 OR #4) AND (#5 OR #6)
Search date	The final definitive search was performed on t17 April 2025 and was last updated on 22 December 2025 before submitting the final revised version.

### 2.2 Search Strategy and Selection Process

The search terms and strategy were formulated by the lead authors (K.S. and M.S.), and confirmed by the other co‐authors, based on the PECO (Population, Exposure, Comparison and Outcome) framework and combined using Boolean operators (OR, AND, NOT). The first author (K.S.) conducted the initial searches across all specified databases to ensure accuracy. Any identified errors were corrected. The search strategy was then refined in collaboration with an expert in systematic reviews (D.L.). The full list of search terms and the final strategy are detailed in Table 1.

The results from each database search were imported into Rayyan (https://www.rayyan.ai), a web‐based collaboration platform that streamline the production of systematic reviews. The software automatically removed duplicate records as the first step (electronic elimination of duplicate records). Following this, the first author (K.S.) conducted a detailed title and abstract screening within the platform, in consultation with the senior author (M.S.). After this screening, the potentially eligible studies were included for full‐text review and subsequent data extraction, based on mutual agreement among the authors.

In cases where potentially included studies were not in English, the reviewers used DeepL, an online translation platform (https://www.deepl.com) to translate them into English, and the corresponding authors were contacted when further clarification was required. When full text articles were not available or lacked sufficient information, to avoid incorrect exclusion, the reviewers posted queries on research community websites (https://www.researchgate.net/) and contacted the corresponding authors directly. Additional studies were identified through reference cross‐checks and supplementary searches to ensure a comprehensive coverage of the evidence.

### 2.3 Data Collection Process and Data Items

Data on the association between multimorbidity/comorbidity and/or polypharmacy and oral health outcomes were extracted from the included studies. The extracted information included study design, participant characteristics, mean age, sample size, definitions of multimorbidity/comorbidity and polypharmacy, and oral health outcome measures. The major findings from each study were summarised focusing on the review's research question.

### 2.4 Study Risk of Bias Assessment

The risk of bias of the included cohort studies was assessed using Newcastle‐Ottawa Scale (NOS) [14]. The included cross‐sectional studies were assessed using NOS adapted for cross‐sectional studies [15].

### 2.5 Summary Measures and Synthesis of Results

A descriptive analysis was conducted to summarise the study design, participant characteristics, exposure definitions, oral health outcomes, and main findings. A meta‐analysis was performed using Review Manager (RevMan) version 5.4.1 (The Nordic Cochrane Center, The Cochrane collaboration, Copenhagen, Denmark). Odds ratio (OR) values along with their respective 95% CIs, were used to explore the association between multimorbidity/polypharmacy and oral health conditions. The Tau‐squared (Tau^2^), Chi‐squared (Chi^2^), and *I*‐squared (*I* ^2^) statistics were used to assess heterogeneity among studies. When appropriate, results were presented using forest plot.

### 2.6 Publication Bias

Publication bias was evaluated using funnel plots when a meta‐analysis included more than 10 studies, in accordance with recommended methodological guidance [16].

## 3 Results

### 3.1 Study Selection

The initial search identified 3433 records (MEDLINE [PubMed]: *n* = 1944; Embase: *n* = 1041; CENTRAL: *n* = 448). The automated removal of duplicates by the software resulted in the elimination of 1118 records. A total of 2876 records were further screened by title and abstract, resulting in 202 potentially included records. Full texts of these 202 records were retrieved and assessed for eligibility, of which 185 records were excluded due to ineligible participants age (*n* = 105), ineligible study design (*n* = 49), reviews (*n* = 18), irrelevant study outcomes (*n* = 11), and study protocols (*n* = 2). In one record, the mean age of participants was unclear. Although the authors were contacted for clarification, the study was eventually excluded due to ineligible participant age [17]. In addition, studies addressing the review questions but not meeting the mean age inclusion criteria were retained for references [18, 19, 20, 21, 22, 23, 24, 25, 26, 27, 28, 29, 30, 31, 32]. No studies evaluating management targeting multimorbidity or polypharmacy with the aim of improving oral health outcomes were identified.

**FIGURE 1:**
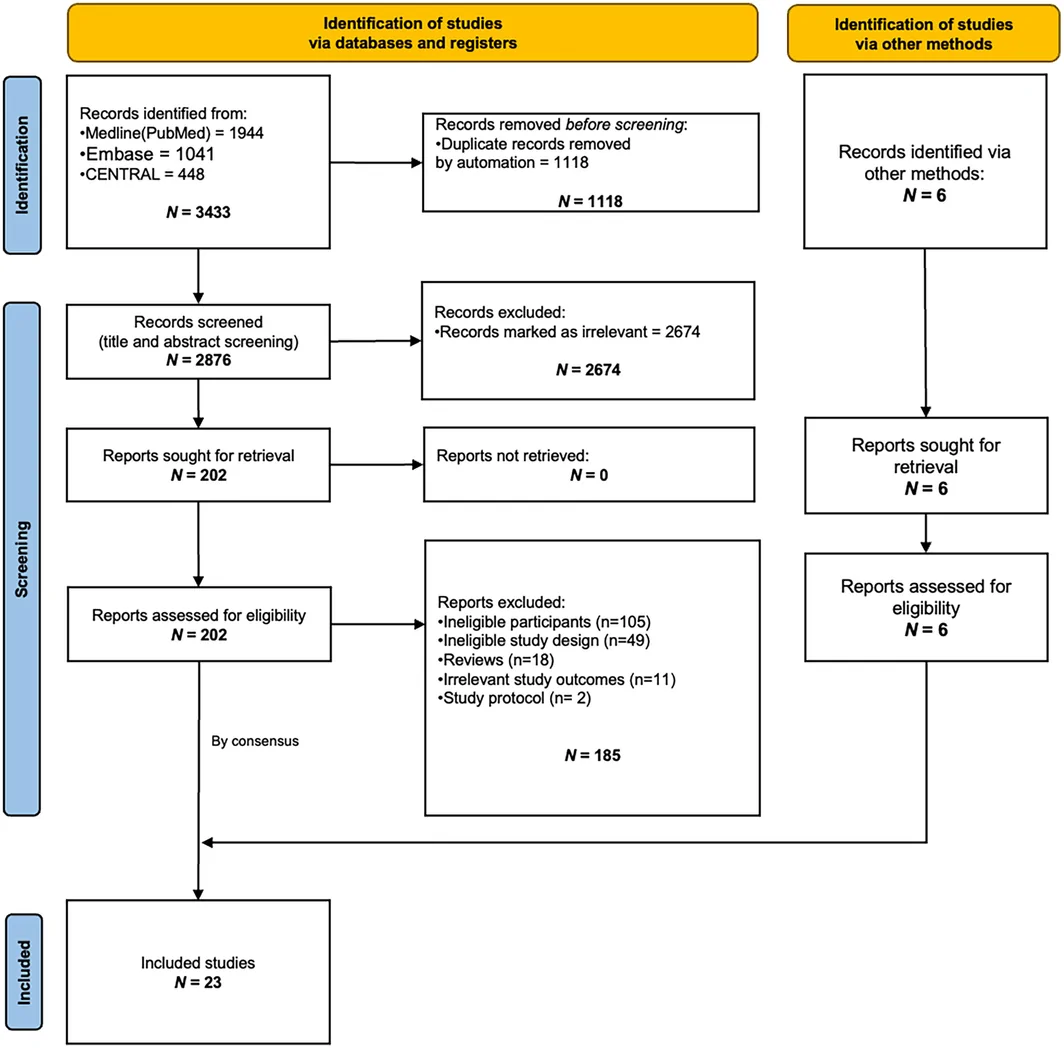
PRISMA flow diagram summarising the study selection process, including records identified, duplicates removed, records screened, and studies included in the meta‐analysis. *N* or *n*, number. [Colour figure can be viewed at wileyonlinelibrary.com]

Finally, total of 23 studies met the inclusion criteria and were included in the analysis: 17 records identified through the systematic search [33, 34, 35, 36, 37, 38, 39, 40, 41, 42, 43, 44, 45, 46, 47, 48, 49] and 6 records identified through additional sources [50, 51, 52, 53, 54, 55]. The step‐by‐step study selection process is summarised in a PRISMA flow diagram (Figure 1). Details of the excluded studies, along with the reasons for their exclusion, are provided in Data S2.

### 3.2 Studies Characteristics

Most of the included studies were cross‐sectional (*n* = 20). Only 2 studies coducted a prospective cohort design, with follow‐up periods of 6 months and 3 years, and 1 study was retrospective. Participants were mainly care‐dependent older adults (12 studies), including those in institutional care (7 studies), hospitalised patients (3 studies), individuals with dementia (1 study), and those requiring home medical care (1 study). While 10 studies were conducted among community‐dwelling older adults, and 1 study included both populations.

Six studies examined the impact of multimorbidity, 9 studies examined polypharmacy, and 8 studies investigated both. Multimorbidity was defined as the presence of two or more diseases in 3 studies and three or more diseases in 1 study. Four studies assessed comorbidity using the CCI, 2 studies used the FCI, and 3 studies examined associations based on the number of diseases. Regarding polypharmacy, 4 studies defined it as the use of five or more medications, 2 studies used a threshold of six or more medications, and 10 studies evaluated associations based on the number of medications. Most studies assessed oral conditions including number of teeth, edentulism, oral function, overall oral health, and dry mouth. All included studies are reported descriptively in Table 2.

**TABLE 2:** Summary of included studies.

Author/year	Study design	Population	Age (Years)	Number of participants (*n*)	Multimorbidity definition	Polypharmacy definition	Focus outcomes	Main findings
Anliker et al. 2023 [50]	Cross‐sectional	Institutionalised older adults	85.5 ± 7.4	180	Two or more diseases	Five or more medications	Number of teeth	The number of teeth was not different between older adults with and without multimorbidity or polypharmacy
Hirata et al. 2025 [51]	Cross‐sectional	Community‐dwelling older adults	78.6 ± 6.9	318	NA	Five or more medications	Oral function (3 items of the Kihon checklist; mastication, swallowing, dry mouth)	Participants with low oral function were more likely to have polypharmacy; however, logistic regression analysis showed that polypharmacy was not significantly associated with low oral function, whereas physical frailty was
Ichikawa et al. 2011 [52]	Cross‐sectional	Community‐dwelling older adults	79–80	368	NA	Number of medications	Stimulated and unstimulated saliva volume	The total number of medications was significantly associated with volumes of both stimulated and unstimulated saliva
Islas‐Granillo et al. 2019 [33]	Cross‐sectional	Institutionalised older adults and community‐dwelling older adults	79.1 ± 9.8	139	Two or more diseases	NA	Functional dentition; edentulism; hyposalivation; dry mouth; root caries; severe periodontitis	The prevalence of edentulism was 55.3% among older adults with multimorbidity compared with 32.7% among those without multimorbidity. Multimorbidity was significantly associated with a higher risk of edentulism, whereas the remaining oral conditions were not significantly associated
Janssens et al. 2017 [53]	Cross‐sectional	Institutionalised older adults	83.9 ± 8.5	1226	NA	Number of medications	Number of teeth; total treatment need; treatment index	A higher number of medications, including xerogenic medications, and risk of dry mouth were associated with a lower number of teeth. In contrast, the total dental treatment need tended to decrease with increasing medication use
Katsoulis et al. 2012 [34]	Retrospective	Older adults attended consultation in a geriatric dentistry clinic	83.7 ± 8.2	192	More than three diseases	NA	Edentulism; number of teeth	Older adults with multimorbidity have higher prevalence of edentulism than those without, but number of teeth were not different
Klotz et al. 2020 [35]	Prospective cohort (6 months follow‐up)	Institutionalised older adults	82.0 ± 9.5	114	Baseline: 5.9 (3.5) diseases. 6 months: 6.5 (3.4) diseases	Baseline: 8.7 (3.7) medications. 6 months: 8.8 (4.2) medications	PI; DHI; OHAT; DMF‐T; decayed teeth; missing teeth; filled teeth	The increasing number of diseases and medications during short‐term evaluation was not associated with all dental variables. While increasing care needs seems to be more associated
Morita et al. 2021 [37]	Cross‐sectional	Community‐dwelling older adults	75 and older	215	NA	Number of medications	Subjective oral function (difficulty eating hard foods, choking or coughing when drinking tea or soup, perceived of dry mouth) and objective oral function (resting saliva assessment, repetitive saliva swallowing test, chewing gum test, Saxon test)	Participants using more than 8 medications was associated with subjective and objective oral dysfunction, with greater medication intake corresponding to more severe oral functional impairment
Nakamura et al. 2021 [54]	Cross‐sectional	Older adults admitted to the recovery and rehabilitation ward	81.9 ± 7.7	471	NA	Six or more medications	OHAT	Polypharmacy was significantly associated with poorer oral health status after adjustment for confounding factors
Ní Chróinín et al. 2016 [38]	Cross‐sectional	Hospitalised older adults	84.4 ± 6.5	202	CCI	NA	OHAT	The CCI was associated with poor oral health status, but not when adjusted for oral pH
Lynge Pedersen et al. 2015 [36]	Cross‐sectional	Community‐dwelling older adults	75.7 ± 6.4	668	Number of systemic diseases	Number of medications	Hyposalivation; oral mucosal lesion; oral candidiasis	The number of medications and systemic diseases was not associated with oral mucosal lesion. Whereas having more than 3 systemic diseases and 6 medications were associated with oral candidiasis
Rosenberg et al. 2021 [39]	Cross‐sectional	Hospitalised older adults in rehabilitation unit	81.9	471	NA	Six or more medications	OHAT	Polypharmacy was significantly associated with poor oral health status
Saarela et al. 2014 [40]	Cross‐sectional	Institutionalised older adults	83.0	1369	CCI	NA	Edentulism	The CCI was not significantly different between edentulous individuals without denture, edentulous individuals with denture, and participants with some natural teeth
Smidt et al. 2011^a^ [41]	Cross‐sectional	Community‐dwelling older adults	75.5 ± 6.4	668	Number of systemic diseases	Number of medications	Dry mouth	The number of systemic diseases and medications was associated with high prevalence of dry mouth
Syrjälä et al. 2011 [42]	Cross‐sectional	Community‐dwelling older adults	79.8 ± 3.6	157	NA	Number of medications	Stimulated and unstimulated salivary flow rate	Participants with low salivary flow rate had higher average number of medications (6.5–7.4) than normal salivary flow rate (4.1–4.3)
Tada et al. 2025 [55]	Cross‐sectional	Institutionalised older adults	75.0 ± 9.1	173	Number of systemic diseases	Number of medications	Dry mouth	The number of disease categories was associated with dry mouth, whereas the number of medications was not significantly associated
Tamaki et al. 2024 [43]	Cross‐sectional	Community‐dwelling older adults	80.0	3222	NA	More than 5 medications	Oral frailty index‐8 (OFI‐8); number of remaining teeth	Polypharmacy was not associated with risk of oral frailty and number of remaining teeth
Tan et al. 2020 [44]	Prospective cohort (3 years follow‐up)	Older adults with dementia using xerogenic medications	80.1 ± 7.6	30,995	CCI	Number of medications	Number of tooth extraction; number of dental restorations	There was no association between the CCI/number of medications and number of tooth or extraction/restoration
Tanaka et al. 2024 [45]	Cross‐sectional	Older adults receiving home medical care	87.0 (83.0–91.0)	93	CCI	NA	OHAT	There was no association between the CCI and OHAT scores
Tuuliainen et al. 2020 [46]	Cross‐sectional	Institutionalised older adults	84.7 ± 5.5	269	FCI	Number of medications	Presence of teeth with dental plaque in 20% or more; presence of teeth with bleeding on probing in 25% or more	There was no association between the FCI or number of medications and presence of dental plaque or bleeding on probing
Viljakainen et al. 2016^a^ [47]	Cross‐sectional	Institutionalised older adults	84.5 ± 5.4	270	FCI	More than 5 medications (10 or more defined as excessive polypharmacy)	Dry mouth	The higher number of systemic diseases and excessive polypharmacy were significantly associated with xerostomia
Weyant et al. 2004 [48]	Cross‐sectional	Community‐dwelling older adults	80.3 ± 4.4	805	NA	Number of medications	Oral function limitation (‘trouble biting or chewing any kind of food like meat or apples’ and whether the ‘kinds/amounts of foods were limited by problems with teeth or dentures’); recency of dental visit	The number of medications was significantly associated with oral function limitations and having a dental visit more than 1 year
Wu et al. 2025 [49]	Cross‐sectional	Community‐dwelling older adults	75.0	3640	Two or more diseases	NA	Having less than 20 teeth	Multimorbidity was associated with having fewer than 20 teeth compared with individuals without chronic diseases

Abbreviations: CCI, Charlson's comorbidity index; DHI, Denture Hygiene Index; DMF‐T, decayed, missing, and filled teeth; FCI, Functional comorbidity index; NA, not applicable; OHAT, Oral Health Assessment Tool; PI, Plaque Index.

^a^ Studies included in meta‐analysis.

### 3.3 Synthesis of Results: Descriptive Analysis of the Included Studies

#### 3.3.1 Number of Teeth and Edentulism

The evidence regarding the association between multimorbidity and number of teeth or edentulism was inconsistent. Studies by Islas‐Granillo et al. [33] and Katsoulis et al. [34] reported that older adults with multimorbidity had a higher prevalence of edentulism compared with those without multimorbidity. Similarly, Wu et al. [49] found an association between multimorbidity and having fewer than 20 remaining teeth. In contrast, Katsoulis et al. and Anliker et al. [50] reported no difference in the number of teeth between older adults with and without multimorbidity. Moreover, studies by Saarela et al. [40] and Tan et al. [44] found no association between the CCI and edentulism (with or without dentures) or number of teeth, respectively.

Regarding polypharmacy, two studies by Anliker et al. and Tamaki et al. [43] reported no significant association between polypharmacy and number of teeth. In contrast, Janssens et al. [53] found that higher medication intake was associated with a lower number of remaining teeth.

#### 3.3.2 Oral Function

Evidence regarding the association between multimorbidity and oral function was not identified, whereas findings on polypharmacy and oral function were inconsistent. Tamaki et al. [43] and Hirata et al. [51] reported no significant association between polypharmacy and the risk of oral frailty, as assessed using the Oral Frailty Index‐8 and the three‐item Kihon Checklist, respectively. In contrast, Weyant et al. [48] observed a relationship between the number of prescribed medications and functional limitations in biting or chewing. Additionally, Morita et al. [37] reported that the use of more than eight medications was associated with both subjective and objective oral dysfunction.

#### 3.3.3 Dry Mouth

Only the study by Islas‐Granillo et al. [33] found that multimorbidity was not associated with either dry mouth or hyposalivation. Similarly, Viljakainen et al. [47] reported that a higher number of diseases was not associated with dry mouth. In contrast, Smidt et al. [41] and Tada et al. [55] found that the number of diseases was associated with dry mouth.

More consistent evidence was observed for the association between polypharmacy and dry mouth. Two studies by Smidt et al. and Viljakainen et al. reported an association between the number of medications and dry mouth, particularly among older adults with excessive polypharmacy (≥ 10 medications). Furthermore, studies by Ichikawa et al. [52] and Syrjälä et al. [42] demonstrated associations between the number of medications and stimulated and unstimulated salivary volume and flow rate, respectively. In contrast, only one study, by Tada et al., reported no difference in the number of medications used by older adults with and without dry mouth.

In addition, Smidt et al. reported that the highest prevalence of dry mouth was observed among patients taking combinations of paracetamol and opioids, psycholeptics and psychoanaleptics, and respiratory medications in combination with other drugs. Both Smidt et al. and Viljakainen et al. similarly reported that individuals with dry mouth were more likely to use specific medication classes, including loop diuretics, proton pump inhibitors, and cardiac agents.

#### 3.3.4 Oral Health and Hygiene

Tanaka et al. [45] and Ní Chróinín et al. [38] reported that the CCI was not associated with oral health status as assessed by the Oral Health Assessment Tool (OHAT). Similarly, Tuuliainen et al. [46] found no significant association between the FCI and OHAT scores. In contrast, studies by Nakamura et al. [54] and Rosenberg et al. [39] reported that polypharmacy was associated with poorer OHAT scores.

Regarding specific clinical oral conditions, Islas‐Granillo et al. [33] found that multimorbidity was not associated with root caries or severe periodontitis, as well as Tuuliainen et al. reported that the FCI was not associated with the presence of dental plaque or bleeding on probing. Interestingly, Janssens et al. [53] observed that the total need for dental treatment tended to decrease with an increasing number of medications. Pedersen et al. [36] found no association between the number of diseases or medications and oral mucosal lesions, but reported that multimorbidity (≥ 3 diseases) and polypharmacy (≥ 6 medications) were associated with oral candidiasis.

Longitudinal evidence suggests that neither multimorbidity nor polypharmacy consistently influences clinical oral health outcomes. In a 3‐year longitudinal study of older adults with dementia receiving xerogenic medications, Tan et al. [44] found no association between CCI or number of medications and the incidence of tooth extractions or restorations. Similarly, Klotz et al. [35] reported that short‐term (6 month) increases in the number of diseases and medications were not associated with changes in OHAT, DMF‐T scores, or general oral health measures. However, Tan et al. also reported that opioid use, specifically, was associated with an increased number of tooth extractions.

### 3.4 Synthesis of Results: Meta‐Analyses of the Outcomes

Four studies provided sufficient information to be included in the meta‐analyses. Two meta‐analyses assessed the associations between multimorbidity and edentulism and dry mouth. Pooled estimates from two studies by Islas‐Granillo et al. [33] and Katsoulis et al. [34] showed a non‐significant association between multimorbidity and edentulism (OR = 1.91, 95% CI: 0.24–15.13), with high heterogeneity (*I* ^2^ = 86%). Similarly, pooled estimates from Islas‐Granillo et al. and Smidt et al. [41] showed a non‐significant association between multimorbidity and dry mouth (OR = 1.87, 95% CI: 0.50–7.00), also with high heterogeneity (*I* ^2^ = 85%). Given the small number of studies, very wide CIs, and substantial heterogeneity, these associations remain inconclusive; therefore, forest plots for these analyses were not presented.

**FIGURE 2:**
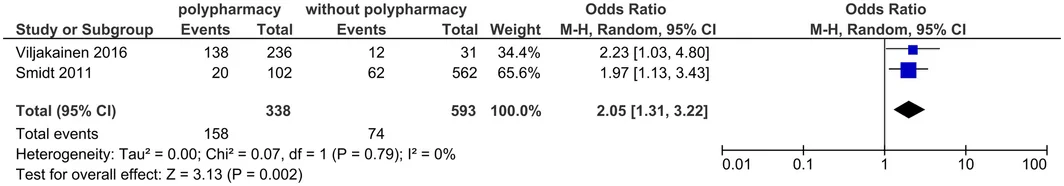
Forest plots of the odd of dry mouth comparing older adults with and without polypharmacy. Chi^2^, Chi‐squared; CI, confidence interval; *I* ^2^, *I*‐squared; M–H, Mantel–Haenzel; Tau^2^, Tau‐squared. [Colour figure can be viewed at wileyonlinelibrary.com]

A meta‐analysis assessed the association between polypharmacy and dry mouth. Two studies by Smidt et al. and Viljakainen et al. [47], both used self‐reported measures of dry mouth. Smidt et al. asked ‘Have you had a daily sensation of dry mouth for more than 3 months?’ while Viljakainen et al. asked ‘Does your mouth feel dry?’ Both studies applied the same definition of polypharmacy (more than 5 medications). The pooled analysis demonstrated a statistically significant association between polypharmacy and dry mouth (OR = 2.05, 95% CI: 1.31–3.22), with no evidence of heterogeneity beyond that expected by chance (*I* ^2^ = 0%) (Figure 2). However, given the small number of included studies, this finding still needs to be interpreted with caution and consideration alongside the narrative synthesis. Assessment of publication bias was not performed due to the small number of included studies.

### 3.5 Risk of Bias and Quality Assessment of the Included Studies

The risk of bias for the included cross‐sectional studies was assessed as predominantly low risk of bias, with 18 of 21 studies, including those incorporated into meta‐analysis. Detailed assessments can be found in Table 3. While the two included cohort studies were rated as moderate risk of bias. Detailed assessments can be found in Table 4.

**TABLE 3:** Risk of bias of included non‐RCTs using Newcastle‐Ottawa scale (adapted for cross‐sectional studies).

First author/year	Selection					Comparability		Outcome			Total (max 10) 0–3 high; 4–6 moderate; 7–10 low
	Representation of exposed cohort	Sample size	Non‐respondents	Ascertainment of exposure (risk factor)		The subjects in different outcome groups are comparable, based on the study design or analysis. Confounding factors are controlled		Assessment of outcome		Statistical test	
Anliker 2023		*		*	*	*		*	*	*	7
Hirata 2025	*	*	*	*	*	*		*		*	8
Ichikawa 2011	*	*		*				*	*	*	6
Islas‐Granillo 2019	*	*	*	*	*	*	*	*	*	*	10
Janssens 2017		*		*		*	*	*	*	*	7
Katsoulis 2012	*	*		*	*			*	*	*	7
Morita 2021	*	*	*	*		*	*	*	*		8
Nakamura 2021		*	*	*	*	*	*	*	*	*	9
Ní Chróinín 2016		*	*	*		*	*	*	*	*	8
Pedersen 2015	*	*	*	*				*	*	*	7
Rosenberg 2021		*		*	*						3
Saarela 2014		*	*	*				*	*	*	6
Smidt 2011	*	*	*	*		*	*	*		*	9
Syrjälä 2011	*	*		*		*	*	*	*	*	8
Tada 2025		*	*	*		*	*	*		*	7
Tamaki 2024	*	*	*	*	*	*	*	*	*	*	10
Tanaka 2024		*	*	*		*	*	*	*	*	8
Tuuliainen 2020		*	*	*		*	*	*		*	7
Viljakainen 2016		*		*	*	*	*	*		*	7
Weyant 2004	*	*		*		*	*	*		*	7
Wu 2025	*	*		*	*	*	*	*	*	*	9

**TABLE 4:** Risk of bias of included non‐RCTs using Newcastle‐Ottawa scale (adapted for cohort studies).

First author/year	Selection				Comparability		Outcome			Total (max 9) 0–3 high; 4–6 moderate; 7–9 low
	Representation of exposed cohort	Selection of non‐exposed cohort	Ascertainment of exposure	Demonstration that outcome of interest was not present at start of study	Comparability of cohorts on the basis of the design or analysis		Assessment of outcome	Was follow‐up long enough for outcome to occur	Adequacy of follow up cohorts	
Klotz 2020			*	*	*	*			*	5
Tan 2020			*		*	*		*	*	5

## 4 Discussion

### 4.1 Principal Findings

This systematic review and meta‐analysis demonstrated a significant association between polypharmacy and dry mouth, as evidenced by the pooled analysis, along with consistent findings linking the number of medications to dry mouth and hyposalivation. In contrast, evidence regarding the association between multimorbidity and dry mouth was inconsistent. Furthermore, polypharmacy was more consistently associated with poorer OHAT scores, whereas findings for comorbidity remained inconsistent. Nevertheless, several studies reported no significant associations between multimorbidity or comorbidity and plaque index, bleeding on probing, root caries, or periodontitis, suggesting that the impact of polypharmacy on oral health conditions may be partly independent of multimorbidity.

The narrative synthesis revealed inconsistent evidence for most oral health outcomes that could not be included in the meta‐analyses due to limited data, particularly regarding the associations of multimorbidity and polypharmacy with edentulism, number of teeth, and oral function. Moreover, longitudinal studies consistently found no associations between either multimorbidity or polypharmacy and outcomes such as tooth extraction, dental restoration, DMF‐T scores, OHAT scores, underscoring the complex and nuanced effects of these exposures on oral health in older populations.

### 4.2 Multimorbidity

Findings from cross‐sectional studies remain inconsistent regarding the impact of multimorbidity or comorbidity on oral conditions. However, longitudinal evidence suggests a more nuanced, bidirectional relationship. A 6‐year longitudinal study found that an increasing number of comorbidities was associated with subsequent tooth loss [20], while a 12‐year longitudinal study showed that edentulism was associated with the progression of multimorbidity, potentially mediated by health behaviours and nutritional factors [56]. This bidirectional relationship may be explained by the progression of multimorbidity leading to declining functional capacity, which can reduce self‐care ability and limit access to oral hygiene practices, ultimately contributing to tooth loss and edentulism [57]. On the other hand, tooth loss, particularly in the absence of dental prostheses, may impair mastication, lead to poorer diet quality, and eventually worsening systemic disease [58].

Multimorbidity may not have a strong direct impact on oral conditions; yet several major geriatric conditions that adversely affect oral health have been highlighted by the WHO and should be considered individually, even in the absence of multimorbidity. These include diabetes, hypertension, rheumatoid arthritis, Alzheimer's disease, Parkinson's disease, and depression [59, 60]. In addition, geriatric medicine recognises the importance of other conditions such as frailty, mobility impairment, sarcopenia, osteoporosis, cognitive impairment, malnutrition, dysphagia, incontinence and sensory impairments (hearing or vision), all of which may indirectly or directly influence oral health [61].

Among these, frailty is a particularly important geriatric syndrome that has demonstrated consistent associations with number of teeth, oral health status, and oral function, and should not be overlooked in the assessment of older adults [62]. The Clinical Frailty Scale (CFS), developed by Rockwood et al., is a widely used 9‐point scale that categorises older adults according to overall fitness and frailty, incorporating elements of comorbidity, cognitive impairment, and functional disability. The CFS provides clinically meaningful predictions of care needs: for example, individuals with well‐controlled medical conditions who are not regularly active are classified as CFS 3, whereas those whose conditions limit activity are classified as CFS 4, representing mild frailty. Higher scores increasingly reflect dependency and care needs, underscoring the importance of frailty assessment when evaluating oral health in older adults together with phisical health [63].

### 4.3 Polypharmacy

According to the evidence, multimorbidity and polypharmacy showed differing associations with oral conditions, suggesting that these two conditions are not inherently related. This distinction is further supported by a meta‐analysis of 65 studies, which reported an inconsistent relationship between multimorbidity and polypharmacy, indicating that they may not always co‐occur [64]. This may be explained by the fact that multiple conditions can be managed using overlapping pharmacological treatments, non‐pharmacological strategies, or deprescribing approaches, particularly in older and frail populations. In contrast, patients suffering from certain systemic diseases tend to experience polypharmacy, including arterial hypertension, coronary artery disease, heart failure, atrial fibrillation, diabetes mellitus, dementia and chronic obstructive pulmonary disease (COPD) [65].

Not only does polypharmacy itself affect dry mouth and hyposalivation, but the number of medications also showed a strong dose‐dependent association with dry mouth. Individuals taking 11 or more, 7–10, or 4–6 medications were 3.34, 2.07 and 1.38 times more likely, respectively, to experience dry mouth compared with subjects who took 0–3 medications [28]. Increasing medication burden is likely associated with a higher anticholinergic load, which can interfere with salivary gland function; however, the mechanisms underlying multidrug interactions contributing to xerostomia have not yet been fully elucidated [66].

Importantly, the effects of individual xerogenic medications should not be overlooked. A meta‐analysis of randomised controlled trials found that urological medications, antidepressants, and psycholeptics were associated with dry mouth in older adults, with the greatest risk observed for medications used to treat urinary incontinence [67]. Moreover, a comprehensive list of medications reported to induce xerostomia and salivary gland hypofunction, categorised by high, moderate and weak levels of evidence, is available in the World Workshop on Oral Medicine report [68].

The observed effects of polypharmacy on oral health may also be reflected in alterations of the oral microbial community. Polypharmacy and anticholinergic burden were inversely associated with microbial diversity and demonstrated significant differences in bacterial community according to polypharmacy status [69]. Additionally, the tooth‐surface commensal *Corynebacterium durum* was reduced in individuals with polypharmacy, while the caries‐associated *Propionibacterium acidifaciens* was increased, independent of differences in salivary flow rates [70]. These changes may indicate oral microbial dysbiosis, which could potentially contribute to the development of oral diseases [71].

### 4.4 Implications for Clinical Practice and Research

Dry mouth should be assessed before treatment planning and monitored closely, particularly after the initiation of new medications. In particular, the use of medications to manage postoperative pain, oral infections, and specific oral lesions should be approached with caution to avoid exacerbating polypharmacy especially in patients with pre‐existing chronic pain [72]. Whenever possible, prescribing decisions should be coordinated with the patient's physician to minimise unnecessary medication burden, with consideration given to non‐pharmacological physical and psychological interventions as alternatives [73]. Cautiously, treatment of certain infections with antiseptic mouthwashes for longer than 2 weeks may worsen xerostomia in patients exposed to polypharmacy [74].

Collaboration between physicians and dentists is essential to balance the therapeutic need for xerogenic medications with efforts to minimise oral dryness symptoms. Notably, prevention of dry mouth should be prioritised, as addressing polypharmacy may help avoid the need for subsequent symptomatic treatment. Optimising medication regimens to effectively manage chronic conditions while minimising the risk of xerostomia is a practical and clinically relevant strategy [75]. Given its modifiable nature, polypharmacy represents an important target for intervention to reduce and potentially prevent dry mouth through medication review and deprescribing. However, randomised controlled trials evaluating the effectiveness of these strategies in reducing xerostomia are currently lacking [76].

Management of medication‐induced xerostomia requires a multimodal approach. Pilocarpine and cevimeline are the most commonly prescribed systemic agents and have been shown to effectively reduce symptoms, although they may cause adverse effects in some patients [77]. Dentists play a crucial role in dry mouth management by focusing on symptom relief and preventive care. Multimodal strategies, including topical therapies, can complement systemic treatments while minimising side effects [78]. Topical agents, such as saliva substitutes or stimulants, can be self‐applied by patients as needed, potentially improving symptomatic relief, although they do not increase salivary flow rate [79].

Future research should place greater emphasis on this specific population, particularly by targeting individuals aged 75 years and older. Well‐designed longitudinal cohort studies are needed to evaluate changes in oral health outcomes over time, especially conditions that develop gradually, such as dry mouth. Studies should also distinguish between short‐term and long‐term effects to better understand disease progression. Furthermore, future investigations should aim to identify specific medication combinations that contribute to oral dryness, helping to clarify whether xerostomia is driven primarily by polypharmacy itself or by particular drug combinations.

### 4.5 Limitations

The majority of the included studies were cross‐sectional, limiting the ability to assess long‐term and causal relationships. In addition, because the inclusion criteria focused on individuals aged 75 years and older, approximately half of the included studies involved institutionalised or hospitalised populations, which may not fully represent the general older adult population due to differences in health status and care needs. There was also considerable heterogeneity in the definitions of multimorbidity and polypharmacy. Many studies assessed exposure based solely, on the number of diseases or medications or, on validated comorbidity indices such as the CCI or FCI, rather than applying common definitions of multimorbidity or polypharmacy. This variability limited the comparability of studies and reduced their suitability for inclusion in the meta‐analyses. Furthermore, the meta‐analyses included a small number of studies and demonstrated substantial heterogeneity, which should be considered when interpreting the pooled results.

## 5 Conclusions

Polypharmacy may impact on dry mouth and hyposalivation in older adults, potentially independent of multimorbidity. However, the impact of multimorbidity and polypharmacy on other oral conditions remains unclear. Further high‐quality evidence, particularly from longitudinal studies, is needed to clarify these relationships and establish causal associations.

## Author Contributions

K.S., S.M., C.R.L., G.M., F.M. and M.S. conceived ideas; K.S. and M.S. collected the data; K.S. and M.S. analysed the data; K.S. and M.S. interpreted the results; K.S. and M.S. led the writing and the preparation of the initial draft. K.S., S.M., C.R.L., G.M., F.M. and M.S. reviewed and edited the manuscript; all authors approved the final version of the manuscript.

## Funding

The authors have nothing to report.

## Ethics Statement

The authors have nothing to report.

## Consent

All authors confirm their participation in the study leading to this manuscript and consent to its submission to be considered for publication.

## Conflicts of Interest

The authors declare no conflicts of interest.

## Supporting information


**Data S1:** PRISMA Checklist.

**Data S2:** Excluded studies with reasons.

## Data Availability

Data sharing not applicable to this article as no datasets were generated or analysed during the current study.
