# Supplementary material for: Associations Between Multimorbidity, Polypharmacy and Oral Conditions in Older Adults: A Systematic Review and Meta‐Analysis
Source: Gerodontology. 2026 May 17;43(3):403–19. doi: 10.1111/ger.70065 (PMC13498933; doi:10.1111/ger.70065)
Supplement: Supplementary file 1 [file GER-43-403-s001.docx]

| **Author** | **Year** | **Title** | **DOI** | **Reasons** |
| --- | --- | --- | --- | --- |
| Ambrosio-Palma | 2022 | Prevalence and Biological Correlates of Oropharyngeal Dysphagia in Outpatients of a Geriatric Evaluation Clinic: A Brief Report | doi:10.1159/000518559 | Ineligible participants |
| Fuentes-García | 2013 | Oral health-related quality of life of older people from three South American cities | doi:10.1111/j.1741-2358.2012.00649.x | Ineligible participants |
| Lam | 2009 | Assessment of the use of xerogenic medications for chronic medical and dental conditions among adult day health participants | doi:10.4140/tcp.n.2009.755 | Ineligible participants |
| Tiisanoja | 2017 | Sedative load, carious teeth and infection in the periodontium among community-dwelling older people | | Ineligible participants |
| Tiisanoja | 2018 | The association of medications with sedative properties with oral health behaviour in community-dwelling older people | doi:10.1111/idh.12285 | Ineligible participants |
| Visser | 2025 | [Important medical-dental interactions. Medication and older people with frailty in the oral healthcare practice] | doi:10.5177/ntvt.2025.02.24115 | review |
| Hamasha | 1998 | Medical conditions associated with missing teeth and edentulism in the institutionalized elderly | doi:10.1111/j.1754-4505.1998.tb00917.x | Ineligible participants |
| Abe | 2022 | Reduced masticatory performance and not using dentures are associated with hypertension in older adults with tooth loss: the Shimane CoHRE study | doi:10.1038/s41440-022-00976-3 LK - | Ineligible participants |
| Abe | 2021 | Number of teeth and masticatory function are associated with sarcopenia and diabetes mellitus status among community-dwelling older adults: A Shimane CoHRE study | doi:10.1371/journal.pone.0252625 LK - | Ineligible participants |
| Casanova-Rosado | 2021 | Association of Edentulism with Various Chronic Diseases in Mexican Elders 60+ Years: Results of a Population-Based Survey | doi:10.3390/healthcare9040404 | Ineligible participants |
| Ajwani | 2003 | Periodontal disease and mortality in an aged population | doi:10.1111/j.1754-4505.2003.tb00297.x LK - | Ineligible participants |
| Aladel | 2023 | General and oral health status and malnutrition among older adults: A cross sectional study | doi:10.1016/j.clnesp.2023.09.835 LK - | Ineligible participants |
| Alghofaili | 2023 | Association of Oral and General Health with Nutritional Status of Older Adults Attending Two Medical Centers in Riyadh, Saudi Arabia: A Cross-Sectional Study | | Ineligible participants |
| Alqahtani | 2022 | Combinations of chronic conditions, functional limitations and geriatric syndromes associated with periodontal disease | | Irrelevant study outcomes |
| Ayrjälä | 2010 | Number of teeth and selected cardiovascular risk factors among elderly people | doi:10.1111/j.1741-2358.2009.00328.x | Irrelevant study outcomes |
| Araujo | 2023 | Functional dentition and edentulism associated with mortality: A cohort study of older adults in Southern Brazil | doi:10.1111/cdoe.12862 LK - | Ineligible participants |
| Barma | 2016 | EVALUATION OF C-REACTIVE PROTEIN AS A POSSIBLE MEASURE OF BIOLOGICAL RESILIENCE IN OLDER PEOPLE | doi:10.1093/ageing/afw033.01 LK - | Ineligible participants |
| Barma | 2016 | Slower Decline in C-Reactive Protein after an Inflammatory Insult Is Associated with Longer Survival in Older Hospitalised Patients | doi:10.1371/journal.pone.0159412 LK - | Ineligible participants |
| Borges | 2010 | Xerostomia and hyposalivation: a preliminary report of their prevalence and associated factors in Brazilian elderly diabetic patients | | Ineligible participants |
| Beltrán | 2023 | Tooth Loss and Caries Experience of Elderly Chileans in the Context of the COVID-19 Pandemic in Five Regions of Chile | doi:10.3390/ijerph20043001 LK - | Ineligible participants |
| Bengtsson | 2021 | Periodontitis related to cardiovascular events and mortality: a long-time longitudinal study | doi:10.1007/s00784-020-03739-x LK - | Ineligible participants |
| Bolerázska and Birková | 2021 | Oral manifestations of pharmacological treatment of systemic diseases | doi:10.36290/far.2021.014 LK - | review |
| Bomfim | 2021 | Multimorbidity and tooth loss: the Brazilian National Health Survey 2019 |  | review |
| Borges-Yáñez | 2006 | Risk factors and prevalence of periodontitis in community-dwelling elders in Mexico | doi:10.1111/j.1600-051X.2006.00897.x LK - - | Ineligible participants |
| Bozdemir | 2016 | General health and oral health status in elderly dental patients in Isparta, Turkey |  | Ineligible participants |
| Cannon | 2023 | From Drugs to Dry Mouth: A Systematic Review Exploring Oral and Psychological Health Conditions Associated with Dry Mouth in Older Adults with Polypharmacy | doi:10.1007/s40266-023-01017-5 LK - | Ineligible participants |
| Fornari | 2021 | Prevalence of xerostomia and its association with systemic diseases and medications in the elderly: a cross-sectional study | doi:10.1590/1516-3180.2020.0616.R3.1902021 | Irrelevant study outcomes |
| Chatzopoulos | 2018 | Systemic medical conditions and periodontal status in older individuals | doi:10.1111/scd.12319 LK - | Irrelevant study outcomes |
| Chellappa and Leelavathi | 2020 | Association of systemic diseases with periodontitis among patients attending a private dental college in Chennai-a record based study | doi:10.37506/ijfmt.v14i4.12384 LK - | Irrelevant study outcomes |
| Chuinsiri and Thinsathid | 2025 | Oral pain and comorbidities in an edentulous older population: A k-prototypes cluster analysis | doi:10.1371/journal.pone.0319819 LK - | Irrelevant study outcomes |
| Coccia | 2015 | Oral health conditions of the elderly in Ancona: A population-based study |  | Ineligible participants |
| Kuroiwa | 2014 | Evaluation of salivary flow and drug interactions in patients with a diagnosis of diabetes mellitus | | Irrelevant study outcomes |
| Da | 2023 | Association between occlusal support and cognitive impairment in older Chinese adults: a community-based study | doi:10.3389/fnagi.2023.1146335 LK - | Ineligible participants |
| Dai | 2022 | Denture wearing status, cardiovascular health profiles, and mortality in edentulous patients: A prospective study with a 27-year follow-up | doi:10.1016/j.jdent.2022.104287 LK - | Ineligible participants |
| Dai | 2023 | Tooth loss, denture use, and all-cause and cause-specific mortality in older adults: a community cohort study | doi:10.3389/fpubh.2023.1194054 LK - | review |
| de Almeida Mello | 2019 | Cross-Country Validation of the Association Between Oral Health and General Health in Community-Dwelling Older Adults | doi:10.1016/j.jamda.2019.02.020 LK - | Ineligible participants |
| De Andrade | 2013 | Factors related to the increase in the negative impact of oral health on quality of life among Brazilian elders | doi:10.1007/s10654-013-9820-0 LK - | Ineligible participants |
| Del Brutto | 2020 | Intracranial atherosclerotic disease and severe tooth loss in community-dwelling older adults | doi:10.46997/REVECUATNEUROL29200039 LK - | Ineligible participants |
| Dibello | 2025 | Oral frailty indicators and cardio- and cerebrovascular diseases in older age: A systematic review | doi:10.1016/j.mad.2024.112010 LK - | cannot retrieve |
| Dibello | 2023 | The Relationship between Oral Health-Related Quality of Life and Body Mass Index in an Older Population from Southern Italy: The Salus in Apulia Study | doi:10.3390/jpm13091300 LK - | Ineligible participants |
| Huang | 2013 | Poor oral health and quality of life in older U.S. adults with diabetes mellitus | doi:10.1111/jgs.12452 | Ineligible study design |
| Dundar and Ilhan Kal | 2007 | Oral mucosal conditions and risk factors among elderly in a Turkish school of dentistry | doi:10.1159/000098415 LK - | review |
| Dean | 2016 | Oral health for adults in care homes | doi:10.7748/ns.31.2.15.s16 | Ineligible participants |
| Fonad | 2015 | Associations between falls and general health, nutrition, dental health and medication use in Swedish home-dwelling people aged 75 years and over | doi:10.1111/hsc.12182 | review |
| Kotronia | 2021 | Poor Oral Health and Inflammatory, Hemostatic, and Cardiac Biomarkers in Older Age: Results From Two Studies in the UK and USA | doi:10.1093/gerona/glaa096 | Ineligible study design |
| Paunovich | 1997 | The most frequently prescribed medications in the elderly and their impact on dental treatment | | Ineligible participants |
| Elter | 1999 | Etiologic models for incident periodontal attachment loss in older adults |  | Ineligible participants |
| Scannapieco and Cantos | 2016 | Oral inflammation and infection, and chronic medical diseases: implications for the elderly |  | Ineligible study design |
| Ferguson | 2020 | Medication taking in a national sample of dependent older people | doi:10.1016/j.sapharm.2019.05.010 LK - | Ineligible participants |
| Ferreira | 2008 | Tooth loss, denture wearing and associated factors among an elderly institutionalised Brazilian population | | Ineligible participants |
| Fredj | 2019 | ORAL HEALTH IN THE ELDERLY PATIENT HOSPITALIZED IN AN INTERNAL MEDICINE DEPARTMENT | doi:10.12890/2019_V6Sup1 LK - | Ineligible participants |
| Herrmann | 2017 | [Xerostomia and its impact on oral health-related quality of life] | doi:10.1007/s00391-015-0968-y | Ineligible participants |
| Manupomé | 2006 | The relationship between cardiovascular xerogenic medication intake and the incidence of crown/root restorations | doi:10.1111/j.1752-7325.2006.tb02551.x | Ineligible participants |
| Geismar | 2006 | Periodontal disease and coronary heart disease | doi:10.1902/jop.2006.050405 LK - | Ineligible participants |
| Hajek | 2023 | Oral health, anxiety symptoms and depressive symptoms: findings from the survey of health | doi:10.1111/psyg.12963 LK - | Ineligible study design |
| Tung and Ford | 2023 | Incident edentulism and number of comorbidities among middle-aged and older Americans | doi:10.1111/ger.12675 | Ineligible participants |
| Lee | 2010 | More missing teeth are associated with poorer general health in the rural Korean elderly | doi:10.1016/j.archger.2009.01.005 | Ineligible participants |
| Holmlund | 2006 | Severity of periodontal disease and number of remaining teeth are related to the prevalence of Myocardial infarction and hypertension in a study based on 4,254 subjects | | Ineligible study design |
| Hong | 2024 | Gender differences in the association between transitions in depressive symptoms and oral health among older adults with chronic conditions | doi:10.1016/j.jad.2024.06.052 LK - | Irrelevant study outcomes |
| Huang | 2012 | Poor oral health and quality of life in U. S. older adults with diabetes | doi:10.1111/j.1532-5415.2012.04000.x LK - | Ineligible participants |
| Hugo | 2009 | Oral status and its association with general quality of life in older independent-living south-Brazilians | doi:10.1111/j.1600-0528.2009.00459.x LK - | Ineligible participants |
| Johansson | 2016 | Oral Health Status of Older Adults in Sweden Receiving Elder Care: Findings From Nursing Assessments | doi:10.1097/NNR.0000000000000158 | Ineligible study design |
| Lewis | 1993 | Use of medications with potential oral adverse drug reactions in community-dwelling elderly | doi:10.1111/j.1754-4505.1993.tb01470.x | Ineligible participants |
| Ilhan | 2015 | Oral health-related quality of life among institutionalized patients after dental rehabilitation | doi:10.1111/ggi.12413 LK - | Ineligible study design |
| Ishii | 2022 | Influence of oral health on frailty in patients with type 2 diabetes aged 75 years or older | doi:10.1186/s12877-022-02841-x LK - | Ineligible study design |
| Islas-Granillo | 2011 | Edentulism risk indicators among Mexican elders 60-year-old and older | doi:10.1016/j.archger.2010.12.014 LK - | Ineligible participants |
| Iwasaki | 2021 | A population-based cross-sectional study of the association between periodontitis and arterial stiffness among the older Japanese population | doi:10.1111/jre.12835 LK - | Ineligible study design |
| Iwasaki | 2012 | Periodontal disease and decreased kidney function in Japanese elderly | doi:10.1053/j.ajkd.2011.08.027 LK - | Ineligible study design |
| Iwasaki | 2016 | Hyposalivation and dietary nutrient intake among community-based older Japanese | doi:10.1111/ggi.12500 LK - | Ineligible participants |
| Jennings | 2015 | Assessing Oral Hygiene in Hospitalized Older Veterans |  | review |
| Lee and Jeong | 2020 | A Population-Based Study on the Association between Periodontal Disease and Major Lifestyle-Related Comorbidities in South Korea: An Elderly Cohort Study from 2002-2015 | doi:10.3390/medicina56110575 | Ineligible participants |
| Johansson | 2020 | Self-reported dry mouth in 50- to 80-year-old Swedes: Longitudinal and cross-sectional population studies | doi:10.1111/joor.12878 LK - | Ineligible participants |
| Iwai | 2024 | Predictive Factors Associated with Future Decline in Swallowing Function among Japanese Older People Aged ≥ 75 Years | doi:10.3390/ijerph21060674 | Ineligible study design |
| Maeda and Mori | 2020 | Poor oral health and mortality in geriatric patients admitted to an acute hospital: an observational study | doi:10.1186/s12877-020-1429-z | review |
| Kailembo | 2016 | Common risk factors and edentulism in adults, aged 50 years and over, in China, Ghana, India and South Africa: results from the WHO Study on global AGEing and adult health (SAGE) | doi:10.1186/s12903-016-0256-2 LK - | Ineligible study design |
| Kakuta | 2025 | Association between oral health-related quality of life and physical frailty among community-dwelling older adults: A 2-year longitudinal study | doi:10.1016/j.tjfa.2024.100008 LK - | Ineligible participants |
| Kandelman | 2008 | Oral health, general health, and quality of life in older people | doi:10.1111/j.1754-4505.2008.00045.x LK - | Ineligible study design |
| Kanungo | 2021 | Association of oral health with multimorbidity among older adults: Findings from the longitudinal ageing study in india, wave-1, 2017–2019 | doi:10.3390/ijerph182312853 LK - | Ineligible study design |
| Kaura Parbhakar | 2022 | The influence of living conditions and individual behaviors on the oral-systemic disease connection: a cross-sectional analysis | doi:10.1111/jphd.12455 LK - | Ineligible participants |
| Khan | 2024 | Self-reported oral health status, edentulism and all-cause mortality risk in 12 809 Australian older adults: a prospective cohort study | | Ineligible participants |
| Khanagar | 2020 | Age-related Oral Changes and Their Impact on Oral Health-related Quality of Life among Frail Elderly Population: A Review | doi:10.5005/jp-journals-10024-2954 LK - | Ineligible participants |
| Koenig | 2024 | Assessing oral health-related quality of life among older people in home-based care - survey results of the InSEMaP study in Germany | doi:10.1186/s12903-024-04500-6 LK - | Ineligible participants |
| Komiyama | 2020 | Dental Status is Associated With Incident Functional Disability in Community-Dwelling Older Japanese: A Prospective Cohort Study Using Propensity Score Matching | doi:10.2188/jea.JE20180203 LK - | Ineligible participants |
| Kossioni and Karkazis | 1999 | Socio-medical condition and oral functional status in an older institutionalised population | doi:10.1111/j.1741-2358.1999.00021.x LK - | Ineligible study design |
| Kotronia | 2021 | Oral health and all-cause cardiovascular disease, and respiratory mortality in older people in the UK and USA | doi:10.1038/s41598-021-95865-z LK - | Ineligible study design |
| Kotronia | 2019 | THE ASSOCIATION OF POOR ORAL HEALTH WITH A RANGE OF INFLAMMATORY MARKERS: RESULTS FROM TWO POPULATION BASED STUDIES OF OLDER PEOPLE IN THE UK AND USA | doi:10.1136/jech-2019-SSMabstracts.25 LK - | review |
| Lim | 2025 | Association Between Geriatric Oral Health Assessment Index and Cardiovascular Disease in Korean Older Adults | doi:10.3961/jpmph.24.569 | Ineligible participants |
| Kurahashi | 2017 | Factors Associated with Number of Present Teeth in Adults in Japanese Urban City | doi:10.2209/tdcpublication.2016-2200 LK - | Ineligible participants |
| Ramírez | 2024 | Factors influencing xerostomia and oral health-related quality of life in polymedicated patients | doi:10.1111/ger.12724 | Ineligible study design |
| Song | 2024 | [Correlation and mechanism between chronic kidney disease and periodontitis based on cross-sectional data] | doi:10.3760/cma.j.cn112144-20230920-00161 | Ineligible study design |
| Lima | 2019 | Oral health complications in Brazilian and French diabetic older people: A comparative study | doi:10.1016/j.archger.2019.103905 LK - | Ineligible participants |
| Lindmark | 2021 | Oral health is essential for quality of life in older adults: A Swedish National Quality Register Study | doi:10.1111/ger.12514 LK - | Ineligible study design |
| Liu | 2021 | Oral health diseases among the older people: a general health perspective | doi:10.31083/jomh.v17i1.316 LK - | Ineligible participants |
| Longman | 1995 | Salivary gland hypofunction in elderly patients attending a xerostomia clinic | doi:10.1111/j.1741-2358.1995.tb00133.x | Ineligible participants |
| Luo | 2021 | Effects of the Co-occurrence of Diabetes Mellitus and Tooth Loss on Cognitive Function | doi:10.2174/1567205019666211223093057 LK - | Ineligible study design |
| Furuta | 2018 | Tooth loss, swallowing dysfunction and mortality in Japanese older adults receiving home care services | | Ineligible study design |
| Iwasaki | 2019 | Effect of chronic kidney disease on progression of clinical attachment loss in older adults: A 4-year cohort study | doi:10.1002/JPER.18-0464 | Ineligible participants |
| Iwasaki | 2018 | Hyposalivation and 10-year all-cause mortality in an elderly Japanese population | doi:10.1111/ger.12319 | Ineligible study design |
| Saito | 2019 | Number of Teeth, Oral Self-care, Eating Speed, and Metabolic Syndrome in an Aged Japanese Population | doi:10.2188/jea.JE20170210 | Ineligible participants |
| Malicka | 2022 | The impact of socioeconomic status, general health and oral health on Health-Related Quality of Life, Oral Health-Related Quality of Life and mental health among Polish older adults | doi:10.1186/s12877-021-02716-7 LK - | protocol |
| Mamedov | 2022 | Relationship between the state of somatic health and diseases of periodontal tissues and teeth in real clinical practice | doi:10.17116/profmed20222502166 LK - | Ineligible participants |
| Mameno | 2024 | Removable denture use, fit, and all-cause mortality in older adults with reduced occlusal support: The OHSAKA study | doi:10.1016/j.prosdent.2024.10.037 LK - | Ineligible study design |
| Manchery | 2022 | Memory decline in older individuals predicts an objective indicator of oral health: findings from the Sydney Memory and Ageing Study | doi:10.1186/s12903-022-02128-y LK - | Ineligible participants |
| Marito | 2022 | The Association of Dietary Intake, Oral Health, and Blood Pressure in Older Adults: A Cross-Sectional Observational Study | doi:10.3390/nu14061279 LK - | Ineligible participants |
| Matsumura | 2003 | Association of dental status with blood pressure and heart rate in 80-year-old Japanese subjects | doi:10.1536/jhj.44.943 LK - | review |
| Matsuyama | 2023 | Causal Effect of Tooth Loss on Cardiovascular Diseases | doi:10.1177/00220345221120164 LK - | Ineligible participants |
| Sukumar | 2024 | Investigating tooth morbidity risks, prevalence, and interventions in tribal setting: a study protocol focused on the Irula community in Tamil Nadu | doi:10.3389/fdmed.2024.1427597 | Ineligible study design |
| Meyerhoefer | 2021 | Dental Care Use, Edentulism, and Systemic Health among Older Adults | doi:10.1177/00220345211019018 LK - | Ineligible participants |
| Mihara | 2020 | Occlusal support predicts tooth loss in older Japanese people | doi:10.1111/cdoe.12515 LK - | Ineligible study design |
| Milagres | 2015 | Oral health status among oldest-old elders and associated factors | doi:10.1007/s10654-015-0072-z LK - | Ineligible participants |
| Mirhosseini | 2024 | The evaluation of clinical factors affecting oral health impacts on the quality of life of Iranian elderly patients visiting dental clinics: A cross-sectional study | doi:10.1111/scd.12980 LK - | Ineligible participants |
| Singh | 2014 | Oral implications of polypharmacy in the elderly | doi:10.1016/j.cden.2014.07.004 | Ineligible study design |
| Fernandes | 2021 | Relationship between polypharmacy, xerostomia, gustatory sensitivity, and swallowing complaints in the elderly: A multidisciplinary approach | doi:10.1111/jtxs.12573 | Ineligible participants |
| Musacchio | 2021 | Tooth retention predicts good physical performance in older adults | doi:10.1371/journal.pone.0255741 LK - | Ineligible participants |
| Nanba | 2023 | Association of the number of remaining teeth with kidney function in community-dwelling healthy older adults: a cross-sectional study | doi:10.5125/jkaoms.2023.49.5.243 LK - | Ineligible participants |
| Noetzel | 2021 | The impact of oral health on prognosis of older multimorbid inpatients: the 6-month follow up MPI oral health study (MPIOH) | doi:10.1007/s41999-020-00427-7 LK - | Ineligible participants |
| Oliveira | 2020 | Edentulism and all-cause mortality among Brazilian older adults: 11-years follow-up | doi:10.1590/1807-3107bor-2020.vol34.0046 LK - | Ineligible participants |
| Ortíz-Barrios | 2019 | The impact of poor oral health on the oral health-related quality of life (OHRQoL) in older adults: the oral health status through a latent class analysis | doi:10.1186/s12903-019-0840-3 LK - | Ineligible participants |
| Holm-Pedersen | 2005 | Dental caries, periodontal disease | doi:10.1111/j.1532-5415.2005.53160.x | Ineligible participants |
| Norlén | 1991 | Relationship between general health, social factors and oral health in women at the age of retirement | | Ineligible study design |
| Peltzer | 2014 | Prevalence of loss of all teeth (Edentulism) and associated factors in older adults in China, Ghana, Mexico, Russia and South Africa | doi:10.3390/ijerph111111308 LK - | Ineligible study design |
| Pengpid and Peltzer | 2018 | The prevalence of edentulism and their related factors in Indonesia 2014/15 |  | Ineligible study design |
| Pengpid and Peltzer | 2020 | Prevalence and correlates of edentulism in a national sample of older adults in Mexico |  | review |
| Pengpid and Peltzer | 2020 | Prevalence and correlates of oral health problems among a nationally representative sample of older adults in Mexico | | review |
| Pescio | 2006 | Clinical epidemiological study of xerostomia in elderly totally edentulous |  | Ineligible study design |
| Eke | 2016 | Periodontitis prevalence in adults ≥ 65 years of age in the USA |  | review |
| Puturidze | 2019 | CORRELATION OF ORAL HEALTH STATUS WITH GENERAL HEALTH IN ELDERLY LIVING AT RESIDENTIAL HOMES IN GEORGIA | | Ineligible study design |
| Xie and Ainamo | 1999 | Association of edentulousness with systemic factors in elderly people living at home | doi:10.1111/j.1600-0528.1999.tb02011.x | Ineligible participants |
| Qi | 2020 | Association between periodontal disease and tooth loss and mortality in an elderly Chinese population | doi:10.1007/s40520-019-01446-6 LK - | Ineligible participants |
| Qian | 2020 | Alveolar bone loss, tooth loss and oral cancer mortality in older patients: A retrospective cohort study | | Ineligible participants |
| Guiglia | 2010 | Aging and oral health: effects in hard and soft tissues | doi:10.2174/138161210790883813 | Ineligible study design |
| R and P | 2023 | Geriatric Phenotypes and Their Impact on Oral Health | doi:10.1016/j.cger.2023.01.004 | Ineligible participants |
| Suzuki | 2015 | Prognosis-related factors concerning oral and general conditions for homebound older adults in Japan | doi:10.1111/ggi.12382 | Ineligible study design |
| Raju | 2021 | Association of tooth loss with morbidity and mortality by diabetes status in older adults: a systematic review | doi:10.1186/s12902-021-00830-6 LK - | review |
| Ramsay | 2017 | ASSOCIATION OF OBJECTIVE AND PERCEIVED NEIGHBOURHOOD CHARACTERISTICS WITH POOR ORAL HEALTH IN OLDER AGE: RESULTS FROM A CROSSSECTIONAL STUDY OF OLDER BRITISH MEN | doi:10.1136/jech-2017-SSMAbstracts.79 LK - | Ineligible participants |
| Randolph | 2001 | Prevalence of tooth loss and dental service use in older Mexican Americans | doi:10.1046/j.1532-5415.2001.49118.x LK - | Ineligible study design |
| Persson | 1998 | Oral health and medical status in dentate low-income older persons | doi:10.1111/j.1754-4505.1998.tb00907.x | Ineligible study design |
| Ren | 2017 | Edentulism and associated factors among community-dwelling middle-aged and elderly adults in China | doi:10.1111/ger.12249 LK - | review |
| Renvert | 2013 | Tooth loss and periodontitis in older individuals: Results from the Swedish National Study on aging and care | doi:10.1902/jop.2012.120378 LK - | Ineligible study design |
| Bailey | 2004 | Persistent oral health problems associated with comorbidity and impaired diet quality in older adults | doi:10.1016/j.jada.2004.05.210 | Ineligible participants |
| Moreira | 2011 | [Spatial risk and factors associated with edentulism among elderly persons in Southeast Brazil] | doi:10.1590/s0102-311x2011001000017 | Ineligible study design |
| Renvert and Persson | 2016 | Treatment of periodontal disease in older adults | doi:10.1111/prd.12130 | Ineligible participants |
| Saberi Hosnijeh | 2023 | CHARACTERISTICS, TREATMENT AND HEALTHCARE UTILISATION OF PATIENTS WITH XEROSTOMIA IN PRIMARY CARE SETTINGS IN THE UNITED KINGDOM | | Ineligible participants |
| Saito | 2017 | Association between dental visits for periodontal treatment and type 2 diabetes mellitus in an elderly Japanese cohort | doi:10.1111/jcpe.12804 LK - | Ineligible participants |
| Saito | 2019 | Associations of number of teeth with medical costs and hospitalization duration in an older Japanese population | doi:10.1111/ggi.13622 LK - | Ineligible participants |
| Scully and Ettinger | 2007 | The influence of systemic diseases on oral health care in older adults |  | Ineligible study design |
| Seki | 2025 | Effects of dental prostheses and occlusal force on adverse health outcomes in community-dwelling older adults lacking functional dentition: The Longitudinal Study of the Tsurugaya Project | doi:10.1016/j.jnha.2025.100534 LK - | Ineligible study design |
| Sfeatcu | 2011 | Aspects of oral and general health among a community center for the underserved |  | Ineligible participants |
| Shah and Sundaram | 2003 | Impact of socio-demographic variables, oral hygiene practices and oral habits on periodontal health status of Indian elderly: a community-based study | | Ineligible participants |
| Shang | 2024 | Teeth, Health, and Mind: Understanding the Interplay of Social Determinants and Cognitive Decline in Older Adults | doi:10.1177/07334648241292960 LK - | Ineligible study design |
| Shimizu | 2023 | Association between periodontitis and chronic kidney disease by functional atherosclerosis status among older Japanese individuals: A cross-sectional study | doi:10.1111/jcpe.13755 LK - | Ineligible study design |
| Shin | 2018 | Number of existing permanent teeth is associated with chronic kidney disease in the elderly Korean population | doi:10.3904/kjim.2016.386 LK - | Ineligible participants |
| Shin | 2018 | Association between the number of teeth and hypertension in a study based on 13,561 participants | | Ineligible participants |
| Singh | 2015 | Oral health among the elderly in 7 Latin American and Caribbean cities, 1999-2000: a cross-sectional study | | Ineligible participants |
| Sinor | 2018 | Main medical illness, oral health complaints and treatment in elderly patients attending Hospital Universiti Sains Malaysia (HUSM) Dental Clinic | | Ineligible participants |
| Sobiesuo | 2025 | Oral Health and Incident Type 2 Diabetes: A Longitudinal Analysis from the British Regional Heart Study | doi:10.2139/ssrn.5128514 LK - | review |
| Solemdal | 2012 | Association between oral health and body cell mass in hospitalised elderly |  | Ineligible participants |
| Stenman | 2012 | Oral health-related quality of life--associations with oral health and conditions in Swedish 70-year-old individuals | | Ineligible participants |
| Sudiono | 2008 | The features of degenerative diseases and their association with the loss of teeth in the elderly of East Jakarta (Indonesia) | | Ineligible participants |
| Shyu | 2024 | Association of oral health with geriatric syndromes and clinical outcomes in hospitalized older adults | doi:10.1016/j.jnha.2024.100385 | Ineligible participants |
| Syrjälä | 2009 | Number of teeth and myocardial infarction and stroke among elderly never smokers | doi:10.1186/1477-5751-8-6 LK - | review |
| Storbeck | 2022 | Dose-dependent association between xerostomia and number of medications among older adults | doi:10.1111/scd.12662 | Ineligible study design |
| Teixeira | 2020 | Relationship between periodontitis and subclinical risk indicators for chronic non-communicable diseases | doi:10.1590/1807-3107bor-2020.vol34.0058 LK - | Ineligible study design |
| Thomson | 1995 | Dental caries experience and use of prescription medications among people aged 60+ in South Australia | | Ineligible study design |
| Tiisanoja | 2011 | Association between sedative drugs and periodontal infection and dental caries among dentate, home-living elders | | Ineligible participants |
| van der Putten and de Baat | 2023 | An Overview of Systemic Health Factors Related to Rapid Oral Health Deterioration among Older People | doi:10.3390/jcm12134306 LK - | Ineligible study design |
| Vemulapalli | 2024 | Prevalence of complete edentulism among US adults 65 years and older: A Behavioral Risk Factor Surveillance System study from 2012 through 2020 | doi:10.1016/j.adaj.2024.02.002 LK - | Ineligible participants |
| Venegas-Sanabria | 2023 | Oral health and self-rated health in community-dwelling older adults in Colombia | doi:10.1186/s12903-023-03401-4 LK - | Ineligible participants |
| Weintraub | 2019 | Factors Associated With Becoming Edentulous in the US Health and Retirement Study | doi:10.1111/jgs.16079 LK - | protocol |
| Wiener and Sambamoorthi | 2014 | Cross-sectional association between the number of missing teeth and cardiovascular disease among adults aged 50 or Older: BRFSS 2010 | doi:10.1155/2014/421567 LK - | Ineligible participants |
| Loesche and Lopatin | 1998 | Interactions between periodontal disease, medical diseases and immunity in the older individual | | review |
| Thomson | 2002 | Is medication a risk factor for dental caries among older people? | doi:10.1034/j.1600-0528.2002.300309.x | Ineligible study design |
| Thomson | 2021 | Xerostomia and polypharmacy among dependent older New Zealanders: a national survey | doi:10.1093/ageing/afaa099 | Ineligible participants |
| Thomson | 2004 | Incidence of periodontal attachment loss over 5 years among older South Australians | doi:10.1111/j.0303-6979.2004.00460.x | Ineligible participants |
| Thomson | 2000 | Medication and dry mouth: findings from a cohort study of older people | doi:10.1111/j.1752-7325.2000.tb03286.x | Ineligible study design |
| Wu | 2023 | Diabetes, Edentulism | doi:10.1177/00220345231155825 LK - | Ineligible study design |
| Chen and Clark | 2011 | Multidimensional risk assessment for tooth loss in a geriatric population with diverse medical and dental backgrounds | doi:10.1111/j.1532-5415.2011.03425.x | Ineligible study design |
| Chen | 2025 | Presumed periodontitis and multimorbidity patterns: a prospective cohort study in the UK Biobank | doi:10.1007/s00784-025-06309-1 | Ineligible participants |
| Ohara | 2016 | Prevalence and factors associated with xerostomia and hyposalivation among community-dwelling older people in Japan | doi:10.1111/ger.12101 | Ineligible participants |
| Ohara | 2011 | Ratio and associated factors of dry mouth among community-dwelling elderly Japanese women | doi:10.1111/j.1447-0594.2010.00647.x | Ineligible study design |
| Ohara | 2022 | Xerostomia as a key predictor of physical frailty among community-dwelling older adults in Japan: a five-year prospective cohort study from The Otassha Study | doi:10.1016/j.archger.2021.104608 | Ineligible participants |
| Zanella | 2016 | Periodontal disease, tooth loss and coronary heart disease assessed by coronary angiography: a cross-sectional observational study | | Ineligible participants |
| Zhang | 2022 | Loss of Teeth and Coronary Heart Disease Mortality in Older Patients | doi:10.6890/IJGE.202210_16(4).0011 LK - | Ineligible study design |
| Zimmermann | 2023 | Interaction of Systemic Morbidity and Oral Health in Ambulatory Patients in Need of Home Care (InSEMaP): An observational study at the sector boundary between dental and general practice care in Germany | doi:10.1136/bmjopen-2022-063685 LK - | protocol |
| Zorawna | 2023 | Relationship of occlusal status with health-related quality of life among older adults in long-term care facilities | doi:10.1111/joor.13431 LK - | Ineligible study design |
